# Anaplastic Large Cell Lymphoma Associated With Breast Implants

**Published:** 2012-01

**Authors:** Shalini Ravi-Kumar, Omid Sanaei, Mohammad Vasef, Ian Rabinowitz, Mohammad Houman Fekrazad

**Affiliations:** 1 University of New Mexico Cancer Center, Albuquerque, NM, USA.; 2 Razi Hospital, Rasht, Iran.

**Keywords:** Anaplastic, Large Cell Lymphoma, Breast, Implant

## Abstract

A forty two years old woman with a history of bilateral breast augmentation for cosmetic reasons was presented for poor healing of the surgical site. Tissue and periprosthetic fluid were removed from the wound site revealing an atypical lymphoid infiltrate. Subsequently the patient developed axillary lymph adenopathy. Excisional biopsy was performed. Flow cytometry was non-diagnostic. She continued to heal poorly and eventually had removal of implant during a simple mastectomy. A nodular area in the breast specimen showed ALK negative anaplastic large cell lymphoma (ALCL). The patient was treated in the private section, with only a pathology consultation being done at our institution (Figures 1-3).

## INTRODUCTION

Anaplastic Large Cell Lymphomas (ALCL) is extremely rare and accounts for approximately 3% of all Non-Hodgkin Lymphomas (NHL). NHLs of the breast account for less than 0.5% of breast cancers and even in this subgroup, they are mostly of the B cell origin (such as diffuse large cell lymphoma, marginal zone B-cell lymphoma and follicular center cell lymphoma). Oddly, even though most cases of primary breast lymphoma are B-cell lymphomas, only 3 cases of B-cell lymphomas associated with breast implants have been reported in literature. T-cell lymphomas in women with breast implants, including two cases of mycosis fungoides and one case of Sézary syndrome arising from the skin overlying intact breast implants, have also been reported. Therefore, the absolute risk of development of ALCL within the breast remains very low. 

Case studies in the Netherlands suggested an 18-fold increased risk of developing ALCL of the breast after breast implantation. However, the absolute risk was very low,

about 0.1 to 0.3 per 100,000 women with prosthesis per year.^   1 ^ A Swedish epidemiological study reviewed 3486 patients who underwent breast implantation between 1965 and 1993 and analyzed the risk of cancer in this population. A two to three fold increase in the risk of lung cancer was discovered but no increase in breast cancers was found in this population. However, this was anticipated as all the women with breast implants in this study were smokers.^   2 ^ The FDA conducted a review of all cases of ALCL associated with breast implants between 1997 and 2010 and only 34 published cases were found. Although the FDA is aware of approximately 60 cases worldwide, many have not been published.^   3^

## CASE REPORT

**Fig. 1:**
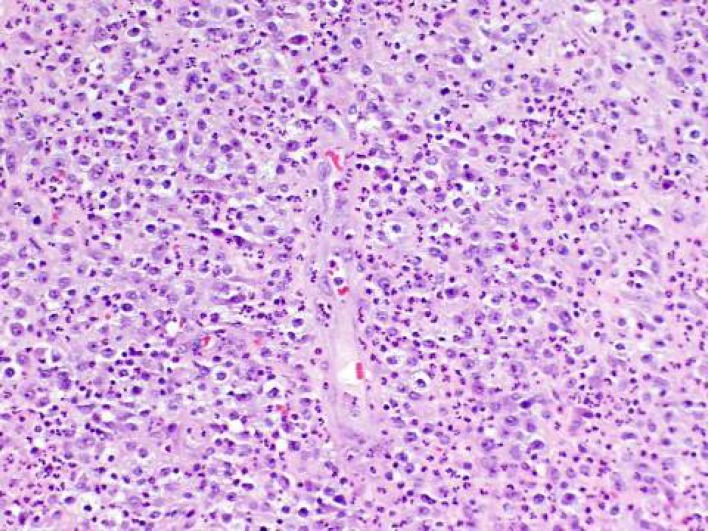
Hematoxylin and eosin stained histologic section of the nodular lesion demonstrate diffuse infiltrate of anaplastic appearing cells with large nuclei, prominent nucleoli, and with moderate amounts of pink cytoplasm consistent with anaplastic large cell lymphoma (ALCL). Notice abundant background neutrophils.

**Fig. 2:**
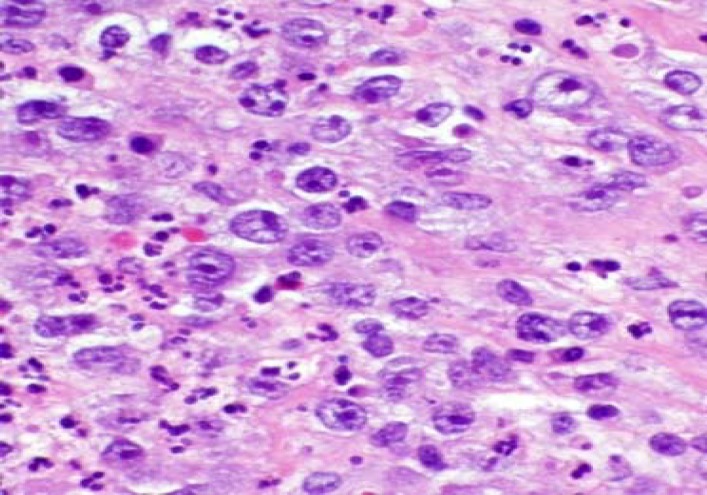
High power view of hematoxylin and eosin-stained section highlights the anaplastic large lymphoma cells with large oval nuclei and prominent nucleoli

**Fig. 3:**
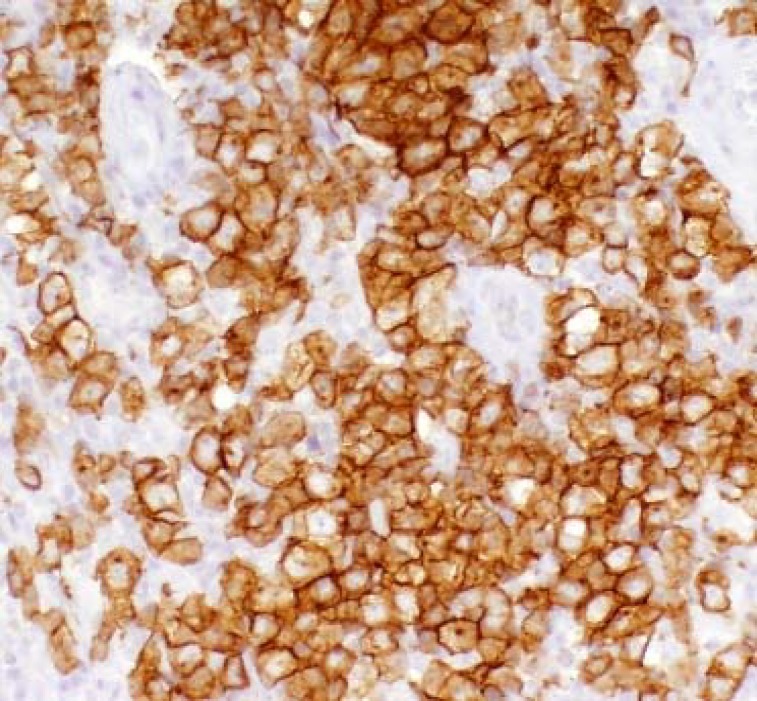
Immunohistochemical stain using CD30 antibody demonstrates uniform and strong expression of CD30 antigen in anaplastic large cells with membrane and Golgi patterns of staining.

A forty two year old woman with a history of bilateral breast augmentation for cosmetic reasons, presented for poor healing of the surgical site. Tissue and periprosthetic fluid removed from the wound site revealed an atypical lymphoid infiltrate (Figure 1). Thereafter, the patient presented with enlargement of an axillary lymph node that was excised. Flow cytometric analysis of the lymph node showed no monoclonal B-cell or aberrant T-cell population and morphology was consistent with a reactive lymph node. The surgical wound continued to heal poorly and therefore a simple mastectomy was performed. A nodular lesion was noted on gross examination of the specimen. The histological section of the nodule showed anaplastic large cell lymphoma (ALCL) which was ALK-negative by immunohistochemical staining (Figures 2 and 3). This case was reviewed and diagnosed by the pathology consultation service at our institution; however, no further information regarding her treatments and outcome is available. 

**EPIDEMIOLOGY**

In the United States, epidemiological studies have not established a higher risk of NHL associated with breast implants. An epidemiological study in the Netherlands between 1990 and 2006 identified 11 women with ALCL of the breast of which five had breast implants. According to this study, the odds ratio was 18.2 (95% CI, 2.1-156.8).^   1 ^ Authors of a Dutch study with 100,000 to 300,000 women with breast implants found an incidence of ALCL of 0.1 to 0.3 per 100,000 women with breast implants per year.^   4 ^ Benjamin Kim *et al.* reviewed the literature of patients with ALCL of the breast to identify if there was an associated seroma. Most reports in their study did not indicate whether the capsule was associated with inflammation or not.^   5 ^

**CLINICAL PRESENTATION**

The age of these patients ranged from 24 to 87 years. Patients usually presented 1-23 years after placement of their breast implants with a mass or a periprosthetic fluid collection. The majority of patients noticed a painful mass. The diagnosis is often established after a core or an excisional biopsy. Analysis of the periprosthetic fluid often showed malignant cells. Alobeid *et al.* reported a case of aggressive ALCL associated with breast implant that was also ALK-1 (Anaplastic lymphoma kinase gene) negative, presenting with bilateral axillary lymph node involvement.^   6^

**DIAGNOSIS**

The diagnosis may be made by cytology of the seroma or capsule biopsy. Positron emission tomography (PET) scan may be helpful as part of the initial work up for some cases. Computed tomography (CT) scans and magnetic resonance imaging (MRI) have also been used. Ultrasound guided aspiration and biopsy are being used in the diagnosis of breast implant associated malignancies. Molecular studies for T and B cell gene rearrangements should be done using the standard polymerase chain reaction (PCR).

It may be challenging to do frequent screening mammograms in women with breast implants as there is a risk of implant rupture. A Japanese study reported that the sensitivity of mammograms in augmented women can be significantly improved by the implant displacement technique. Radiolucent breast implants had some complications and have not been used very much.^   7 ^

**CHARACTERISTICS OF ANAPLASTIC LARGE CELL LYMPHOMA**

Histopathologically ALCL is characterized by large pleomorphic cells with a horse shoe nucleus. The morphology of cells in ALCL of the breast is the same in patients with or without breast implants. These cells have a high mitotic index. About 60 to 85% of these ALCL tumors stain for surface marker CD30 (Ki-1). They are positive for EMA (epithelial membrane antigen) and for other T cell markers such as CD3, CD4, CD5, CD7 or CD43. It is interesting that most systemic ALCL are ALK positive; however, the cases associated with breast implants are ALK negative similar to the cutaneous ALCL presentation.^   8 ^ Both the systemic form and the cutaneous form express CD30, a tumor necrosis factor and a tumor marker found in Hodgkins and ALCLs.^   5 ^ ALK is due to a chromosome translocation t(2;5) (p23;q35). The ALK gene on chromosome 2 is fused to nucleoplasmin gene (NPM) on chromosome 5. The fusion oncoprotein can be detected by a cytoplasmic and nuclear staining pattern by immunochemistry. The systemic ALK negative ALCL have a worse prognosis with 40% 5-year survival as compared with systemic ALK positive ALCL which is 80%. Patients with systemic ALK positive ALCL are younger, whereas patients with systemic ALK negative form are usually older. The ALCL associated with breast implants has the clinical behavior of the primary cutaneous ALCL and is less aggressive. The 5 and 10 year survival rates of these patients are greater than 85%.^   9 ^ Experts believe that ALCL associated with breast implants present in a majority of women as tumors localized to the breast capsule or the seroma and have a clinical course similar to the primary cutaneous ALCL. Only one death has been reported among patients with implant-associated ALCL.^   10 ^ This is not similar to the extranodal ALK-negative ALCL, which is associated with a very poor outcome. 

A review of all cases of ALCL of the breast seen at the MD Anderson Cancer Center over 21 years was published in 2008. Six cases were identified; two of them had a history of cutaneous ALCL and subsequently developed ALK-negative ALCL of the breast. Two other patients had associated breast implants and these were ALK-negative. The other two patient developed ALK-positive ALCL of the breast as a part of their stage IV disease.^   11^

**TREATMENT OF ALCL-ASSOCIATED WITH BREAST IMPLANTS**

CHOP chemotherapy and local irradiation are recommended. Other combination chemotherapies such as daunorubicin, cyclophosphamide, prednisone, vincristine, and prednisone along with etoposide, carboplatin and ifosfamide have been used in these patients. Most of these patients never developped recurrence, however it has been reported. Most of these patients underwent capsulectemy since tumor cells are usually localized to the capsule or seroma. Capsulectemy may be necessary for diagnostic purposes as well. 

**PROSTHESIS TYPE AND PATHOGENESIS**

Breast implants have been used extensively since early 1960s. Of the thirty four cases reported in the literature, twenty four were silicone breast implants, seven were saline breast implants, and three were not specified. Almost 80% of these were used for cosmetic reasons with the rest being for patients who were getting breast reconstruction after mastectomy. It is unlikely that the mechanism of development of ALCL is related to the chemotherapy. Both saline-filled and silicone breast implants have been implicated in the pathogenesis of ALCL of the breast. Although silicone prostheses were thought to be biologically inert, there are case reports with localized and distant granulomatous inflammation in the breast and reactive lymphadenopathy. Animal studies have shown that silicone may be immunogenic. Silicone gels contain potentially active biological compounds such as residual vinyl groups and platinum. Silicone gels can seep out of the capsules or rupture. Rupture has not been reported in many of these ALCL associated with breast implants, but the theory that chronic leakage may play a role is supported by the finding of intracellular macrophages and extracellular silicone in the superficial portion of the periprosthetic breast capsule. Silicone may stimulate T cells chronically leading to a clonal expansion. The three hypotheses proposed to explain the associations of ALCL with breast implants are: direct immunological drive, indirect cytokine mediated drive and toxin damage by silicone products. The silicone products may have a direct oncogenic mechanism.^   9^

Since 1992, in the United States, silicone-filled implants were banned, however saline-filled silicone covered implants continued to be used. Among the 30 cases of breast implant associated ALCL, 22 were associated with the saline based implant (Table 1). 

**Table 1:** Clinicopathological features of implant associated ALCL

Age	Reason for breast implant	Seroma associated	Time to development of ALCL	Side of implant	Side of ALCL	Prosthesis	Tumor size	ALK status	Implant removed?	Reference
65	N/A	Yes	N/A	Left	Left	N/A	Not detected	Positive	N/A	^11^
45	Cancer	Yes	7	Right	Right	Saline	Not detected	Positive	Partially	^9^
59	Cancer	Yes	3	Left	Left	Silicone	N/A	Positive	Yes	^9^
34	Cosmetic	Yes	4	Bilateral	Left	Saline	N/A	Positive	Yes	^9^
44	Cosmetic	Yes	N/A	Left	Left	Saline	N/A	Positive	Yes	Roden 2008
33	Cosmetic	Yes	9	Left	Left	Silicone	1mm	Positive	Yes	^13^
87	Cancer	Yes	8	Right	N/A	Saline	N/A	Positive	N/A	^14^
52	Cosmetic	Yes	14	Right	Right	Silicone	3 cm	Positive	Yes	^15^Newman 2008
64	Cancer	Yes	7	Right	N/A	Saline	N/A	Negative	N/A	^16^
40	Cancer	No	9	Left	Left	Silicone	1.5 cm	Negative	N/A	^11^ ^11^
41	Cosmetic	No	5	Left	Right	Saline	2 cm	Negative	N/A	^17^Keech 1997
40	Cosmetic	No	19	Right	Right	Silicone	0.2 cm	Negative	Yes	^18^
53	Cosmetic	N/A	1	N/A	Left	Rofil Pip hydrogel	N/A	Negative	N/A	^19^
49	Cosmetic	N/A	23	N/A	Bilateral	Silicone	N/A	Negative	Bilateral replacements	^19^
43	Cosmetic	N/A	13	N/A	Right	Silicone	N/A	Negative	Yes	^19^
38	Cosmetic	N/A	13	N/A	Right	Silicone	N/A	Negative	Bilateral replacements	^19^Jama
29	Cosmetic	N/A	3	N/A	Right	Silicone	N/A	Negative	N/A	^19^
58	Breast cancer	Seroma	6	Left	Left	Saline filled silicone	N/A	Negative	Yes	^8^
66	Carcinoma	N/A	12	Left	Left	Saline	N/A	Negative	Yes	^20^
68	Breast cancer	N/A	16	Right	Right	Silicone	N/A	Negative	Yes	^6^Alobeid 2009
68	Breast cancer	Seroma	17	Bilateral	Left	Silicone	N/A	N/A	Yes	^21^

N/A: Not available.

**ESTABLISHMENT OF THE TLBR CELL LINE**

T cell lymphoma breast-1 (TLBR-1) was established from the primary tumor tissue which has the phenotype and cytogenetic of the original ALK negative breast implant associated ALCL. These cells had polymorphic cell shape, enlarged nuclei and nucleoli with abundant cytoplasm. Transplantation of the cultured TLBR-1 cells produces tumors in mice with the same phenotypic features of ALCL. In this case, neither the original tumor, nor the TLBR-1 expressed the ALK protein or contained the associated t(2;5). EMA was decreased in the cytospin and heterotransplant compared with original tissue biopsy. TLBR was strongly positive for CD30, CD26, CD4 and CD2. 

The TLBR-1 cells have significant chromosomal abnormalities including: Partial trisomy 2, an addition involving 5p, a deletion involving 10p, monosomy 16 and 20, and a gain of 2 marker chromosomes. PCR analysis of the TLBR-1 showed TCR Gamma monoclonality and IgH gene rearrangements.TLBR-1 cells showed a statistically significant reduction in tumor suppressor gene expression p53 and RB relative to normal cells. T-ALL associated oncogenes were not up regulated in the TLBR- 1 cell line.^   12 ^

## CONFLICT OF INTEREST

The authors declare no conflict of interest.
